# MAIT cell counts are associated with the risk of hospitalization in COPD

**DOI:** 10.1186/s12931-022-02045-2

**Published:** 2022-05-18

**Authors:** Terezia Pincikova, Tiphaine Parrot, Lena Hjelte, Marieann Högman, Karin Lisspers, Björn Ställberg, Christer Janson, Andrei Malinovschi, Johan K. Sandberg

**Affiliations:** 1 Department of Medical Sciences, Respiratory, Allergy and Sleep Research, Uppsala University, Uppsala, Sweden; 2 Division of Pediatrics, Department of Clinical Science, Intervention and Technology, Karolinska Institutet, Stockholm, Sweden; 3 Center for Infectious Medicine, Department of Medicine, Karolinska Institutet, Stockholm, Sweden; 4 Stockholm CF Center, Karolinska University Hospital Huddinge, Stockholm, Sweden; 5 Department of Public Health and Caring Sciences, Family Medicine and Preventive Medicine, Uppsala University, Uppsala, Sweden; 6 Department of Medical Sciences, Clinical Physiology, Uppsala University, Uppsala, Sweden; 7 Department of Respiratory Medicine and Allergy, K85, Karolinska University Hospital Huddinge, 141 86 Stockholm, Sweden

**Keywords:** Human, COPD, MAIT cells, T cells, Immune activation

## Abstract

**Background:**

Chronic obstructive pulmonary disease (COPD) is characterized by persistent airflow limitation associated with chronic inflammation in the airways. Mucosal-associated invariant T (MAIT) cells are unconventional, innate-like T cells highly abundant in mucosal tissues including the lung. We hypothesized that the characteristics of MAIT cells in circulation may be prospectively associated with COPD morbidity.

**Methods:**

COPD subjects (n = 61) from the Tools for Identifying Exacerbations (TIE) study were recruited when in stable condition. At study entry, forced expiratory volume in 1 s (FEV_1_) was measured and peripheral blood mononuclear cells were cryopreserved for later analysis by flow cytometry. Patients were followed for 3 years to record clinically meaningful outcomes.

**Results:**

Patients who required hospitalization at one or more occasions during the 3-year follow-up (n = 21) had lower MAIT cell counts in peripheral blood at study inclusion, compared with patients who did not get hospitalized (p = 0.036). In contrast, hospitalized and never hospitalized patients did not differ in CD8 or CD4 T cell counts (p = 0.482 and p = 0.221, respectively). Moreover, MAIT cells in hospitalized subjects showed a more activated phenotype with higher CD38 expression (p = 0.014), and there was a trend towards higher LAG-3 expression (p = 0.052). Conventional CD4 and CD8 T cells were similar between the groups. Next we performed multi-variable logistic regression analysis with hospitalizations as dependent variable, and FEV_1_, GOLD 2017 group, and quantity or activation of MAIT and conventional T cells as independent variables. MAIT cell count, CD38 expression on MAIT cells, and LAG-3 expression on both MAIT and CD8 T cells were all independently associated with the risk of hospitalization.

**Conclusions:**

These findings suggest that MAIT cells might reflect a novel, FEV_1_-independent immunological dimension in the complexity of COPD. The potential implication of MAIT cells in COPD pathogenesis and MAIT cells’ prognostic potential deserve further investigation.

**Supplementary Information:**

The online version contains supplementary material available at 10.1186/s12931-022-02045-2.

## Background

Chronic obstructive pulmonary disease (COPD) is a major cause of morbidity worldwide [1]. It is characterized by persistent airflow limitation associated with a chronic inflammatory response in the airways. It is believed that the inflammatory response in COPD lung is driven mainly by CD8 T cells, Th1 cells, and oligoclonal B cells [2]. The Global Initiative for Chronic Obstructive Lung Disease (GOLD) classifications predict COPD hospitalizations and all-cause mortality [3]. The most reliable currently available outcome measure is forced expiratory volume in 1 s (FEV_1_) [4]. However, the prognostic utility of FEV_1_ is limited, and may not reflect the full complexity of COPD [5, 6]. To date no reliable associations with components of cellular immunity have been established to function as candidate biomarkers in COPD.

Mucosal-associated invariant T (MAIT) cells are a subset of unconventional, innate-like T cells relatively abundant in lung, liver and peripheral blood [7, 8]. MAIT cells express a semi-invariant T-cell receptor and recognize microbial-derived metabolites presented by the evolutionarily highly conserved and nonpolymorphic MHC-Ib-related protein 1 (MR1) [9]. The most well-described MR1-presented antigens recognized by MAIT cells are derivatives from the riboflavin pathway, expressed by important pulmonary pathogens such as Pseudomonas aeruginosa and Klebsiella pneumoniae [10, 11]. MAIT cells play an important role in immune defense and homeostasis at mucosal barrier sites and contribute to control of microbial infections of the lung in murine models [12–14]. Furthermore, MAIT cells have a strong tissue homing capacity [15], and display an IL-17-biased pro-inflammatory profile in mucosal tissues [16, 17]. Several chronic and acute conditions are associated with decline of MAIT cells in circulation, sometimes as a result of accumulation at the site of infection or inflammation. This includes diverse inflammatory diseases such as diabetes [18], viral hepatitis [19], and COVID-19 [20, 21]. MAIT cells thus represent a conserved T cell population in humans, exhibit mucosal tissue-homing characteristics, with known roles in bacterial infections of the lung.

COPD has previously been associated with an increased frequency of cytokine-producing CD8 T cells in blood [22], while the circulating MAIT cell frequency was found to be decreased [23]. However, the role of MAIT cells in COPD is largely unknown [24]. Here, based on what we know about the role of MAIT cells in the lungs, we hypothesized that MAIT cell levels and characteristics may be associated with COPD morbidity.

## Methods

### Patient cohort, recruitment and definitions

We recruited COPD subjects (n = 61) from the Tools for Identifying Exacerbations (TIE) study in Uppsala [25]. Patients from primary and secondary care settings with a diagnosis of COPD (ICD code J44.0, J44.1, J44.8 and J44.9) and smoking history ≥ 10 pack-years were included. The diagnosis was confirmed at the inclusion visit with spirometry using the post-bronchodilator (400 mcg salbutamol) FEV_1_ divided by the highest vital capacity value from a slow or forced maneuver with a ratio of < 0.70 (SpiroPerfect spirometer, Welch Allyn, Skaneateles Falls, NY, USA or Jaeger Masterscreen PFT, Erich Jaeger GmbH, Wurzburg, Germany). At inclusion, all participants were in stable clinical condition. At study entry, postbronchodilator FEV_1_, COPD Assessment Test (CAT) and current medication lists were recorded, and peripheral blood mononuclear cells (PBMCs) were isolated and cryopreserved for later analysis by flow cytometry. The subjects were followed prospectively for 3 years. At the end of the follow-up period, the patients were classified according to GOLD 2017 guidelines [26] and clinically meaningful outcomes were recorded (Table 1).

**Table 1:** Clinical characteristics of the COPD cohort, based on data collected at inclusion or during follow-up

Study participants	All (n = 61)	Never hospitalized (n = 40)	Hospitalized (n = 21)	p-value
Female/Male, n (%)	35 (57)/26 (43)	24 (60)/16 (40)	11 (52)/10 (48)	0.597
Age at inclusion, median (range), years	70 (55–76)	70 (55–75)	69 (56–76)	0.701
Smoking exposure at inclusion, packyears	38 (25–44)	40 (25–45)	37 (23–40)	0.324
Current smoker/Ex-smoker at inclusion, n (%)	13 (23)/43 (77)	7 (20)/28 (80)	6 (29)/15 (71)	0.523
Baseline FEV_1_, % predicted	57 (47–69)	62 (50–70)	51 (40–63)	0.039
Baseline FEV_1_/FVC, ratio %	49 (40–57)	50 (41–57)	44 (36–57)	0.307
Baseline CAT score	12 (9–16)	11 (8–16)	14 (10–17)	0.074
Baseline CAT ≥ 10/CAT < 10, n (%)	42 (69)/19 (31)	26 (65)/14 (35)	16 (76)/5 (24)	0.561
Baseline BMI, median (range)	25 (17–38)	25 (18–38)	26 (17–35)	0.703
Inhaled corticosteroids at inclusion, Y/N, n (%)	35 (57)/26 (43)	18 (45)/22 (55)	17 (81)/4 (19)	0.013
CRP at inclusion (mg/L)	3.0 (1.4–4.2)	2.9 (1.3–3.7)	3.7 (1.7–5.9)	0.074
Leucocyte count at inclusion (× 10^9^/L)	7.1 (6.0–8.4)	6.6 (5.8–8.2)	7.5 (6.7–9.6)	0.019
Heart failure diagnosis at inclusion, Y/N, n (%)	15 (25)/46 (75)	7 (17.5)/33 (82.5)	8 (38)/13 (62)	0.117
Diabetes mellitus at inclusion, Y/N, n (%)	11 (18)/50 (82)	5 (12.5)/35 (87.5)	6 (29)/15 (71)	0.164
Frequent exacerbator, Y/N, n (%)	10 (16)/51 (84)	2 (5)/38 (95)	8 (38)/13 (62)	0.002
GOLD 2017 group, A/B/C/D, n	18/33/1/9	14/24/0/2	4/9/1/7	–
Death or respiratory insufficiency within 3 years, Y/N, n (%)	10 (16)/51 (84)	1 (2.5)/39 (97.5)	9 (43)/12 (57)	0.0001
Received antibiotics within 3 years, Y/N, n (%)	38 (62)/23 (38)	18 (45)/22 (55)	20 (95)/1 (5)	0.0001
Acute exacerbation(s) within 3 years, Y/N, n (%)	39 (64)/22 (36)	21 (52.5)/19 (47.5)	18 (86)/3 (14)	0.012
Total acute exacerbation days within 3 years, median (range)	11 (0–140)	6 (0–89)	20 (0–140)	0.004
Total hospitalization days within 3 years, median (range)	0 (0–112)	0 (0–0)	9 (1–112)	0.00000
Total days on antibiotics per os, median (range)	10 (0–145)	0 (0–35)	35 (0–145)	0.00001
Total days on intravenous antibiotics, median (range)	0 (0–34)	0 (0–0)	3 (0–34)	0.00001

Values are medians with interquartile ranges, unless stated otherwise. Percentages are of those with valid data

For comparison of groups Mann–Whitney U Test was used, for comparing proportions, Fisher’s Exact Test was used

Heart failure was defined according to the patient records; diagnosed by the clinical picture and/or heart echocardiography

Frequent exacerbator was defined as a patient with 2 or more acute exacerbations per year during the 3 years of follow-up (counted as total number of acute exacerbations during the 3 follow-up years divided by 3 years)

Respiratory insufficiency was defined as onset of need of long-term oxygen treatment or need of long-term non-invasive ventilation

Acute exacerbation was defined as an episode of acutely or sub-acutely worsened dyspnea

*BMI* body mass index, *CAT* chronic obstructive pulmonary disease assessment test, *FEV*_*1*_ forced expiratory volume in 1 s, *FVC* forced vital capacity, *GOLD* Global Initiative for Chronic Obstructive Lung Disease

Participants aged 40–80 years with established COPD diagnosis were eligible. Exclusion criteria included established diagnosis of asthma, the asthma–chronic obstructive pulmonary disease overlap syndrome (ACOS), speech difficulties, dementia, psychotic disorders, and severe comorbidities associated with expected survival less than 12 months. In addition, because of known bad prognosis, patients with ongoing long-term oxygen treatment were not eligible for inclusion into the study. At inclusion and time of sampling, all participants were in stable clinical condition regarding their COPD disease, and at least four weeks had passed since their latest acute exacerbation.

To assess lung function, we measured postbronchodilator (400 µg salbutamol) dynamic spirometry volumes in liters at inclusion. FEV_1_ percentage of predicted (% predicted) values were calculated using Hedenström reference equation [27, 28].

Based on COPD Assessment Test (CAT) scores and exacerbation frequency during the 3 years of prospective follow-up, the patients were classified according to the GOLD 2017 guidelines into groups A, B, C and D [26]. Exacerbation frequency was counted as mean number of exacerbations per year (i.e., the total number of exacerbations during the 3 years, divided by 3 years). Exacerbation was defined as an acute or sub-acute worsening of dyspnea.

We aimed to investigate if MAIT cells may be associated with COPD morbidity. As a measure of morbidity, we chose all-cause hospitalization during the 3-year follow-up because it is a strictly objective outcome that is easily measured, associated with considerable healthcare costs and strongly linked to mortality [29].

### Flow cytometry analyses and monoclonal antibodies

PBMCs were isolated from 30 ml heparinized blood using the Ficoll-Paque (Lymphoprep, Axis-Shield), and cryopreserved in liquid nitrogen. The PBMCs were thawed, washed, and counts and viability were assessed using the Countess II automated cell counter (Thermo Fisher Scientific). 1 × 10^6^ cells were stained in the dark in a 96-well V-bottom plate. The following antibodies were used for the staining: anti-human CD56 BUV737 (NCAM16.2), CD14 V500 (M5E2), CD19 V500 (HIB19), CCR5 PeCy5 (2D7/CCR5), CD3 AF700 (UCHT1), CD3 BUV805 (UCHT1), Ki-67 BUV395 (B56), Granulysin AF488 (RB1), TCF1 PE (S33-966), PLZF PE-CF594 (R17-809), CD4 APC-H7 (SK3) from BD Biosciences, CD8 BV570 (RPA-T8), CD161 BV605 (NKR-P1A), T-bet BV711 (4B10), Vα7.2-PE-Cy7 (3C10), CD38 BV421 (HIT2), PD1 BV785 (EH12.2H7), HLA-DR APC-H7 (L243) from Biolegend, TIM3 AF488 (344823), TIGIT PE (741182) from R&D systems, and LAG-3 APC (3DS223H) from eBioscience (see Additional file 1: Table S1 for staining panels). The LIVE/DEAD Fixable Aqua dead cell stain kit (ThermoFisher) was used to discriminate dead cells. Cells were stained for 20 min at 4 °C, washed and fixed in BD CellFIX for 10 min at room temperature before acquisition. For the intranuclear panel, cells were fixed and permeabilized 30 min at 4 °C after the surface staining using the Transcription Factor Buffer Set (BD Biosciences), and then stained intracellularly for 30 min at 4 °C. Cells were fixed in BD CellFIX 1X (BD Biosciences) for 10 min at room temperature before acquisition. Samples were acquired on the BD FACSymphony instrument (BD Biosciences) and analyzed with FlowJo version 10.6.2 (Tree Star, Ashland, OR).

**Fig. 1:**
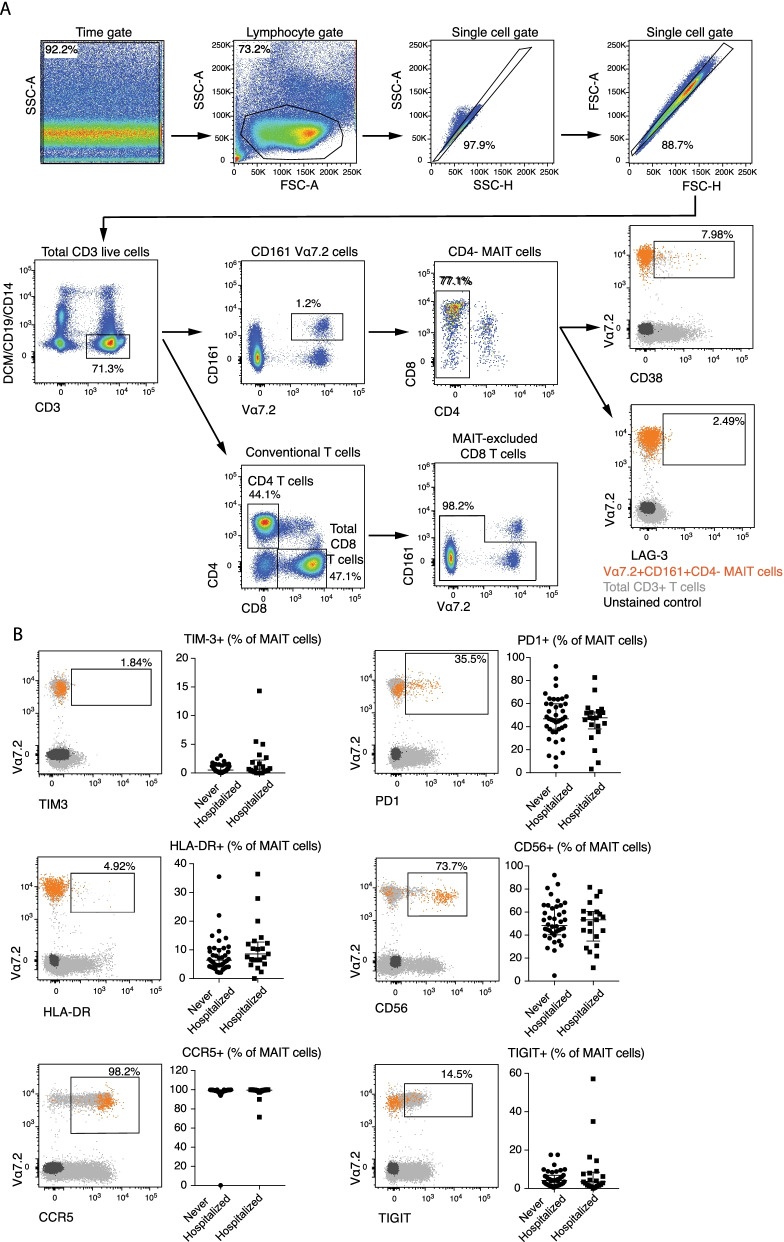
Gating strategy.** A** Gating strategy to identify peripheral blood CD4 T cells, classical CD8 T cells, MAIT cells and expression of the activation markers CD38 and LAG-3 on MAIT cells. Gates for CD38 and LAG-3 expression on MAIT cells (orange) were set based on the expression on total T cells (in grey) when a clear negative and positive population could be identified or based on the unstained control (black) otherwise. MAIT cells were identified as CD4-Vα7.2 + CD161 + T cells. For quantification of classical CD8 T cells, MAIT cells were gated out. **B** Phenotypic comparison of MAIT cells in COPD patients requiring hospitalization or not during the 3-year follow-up. Representative flow cytometry staining of TIM-3, PD1, HLA-DR, CD56, CCR5 and TIGIT on MAIT cells (orange) using the unstained control (black) or the total T cells (grey) as controls to set the gate. The scatter plots display the expression of each marker (median ± IQR) on MAIT cells between Hospitalized (n = 21) and Never Hospitalized (n = 40) COPD patients during the 3-year follow-up. The Mann–Whitney U Test was used to evaluate statistical differences between groups

After gating on lymphocytes, non-single cells and dead cells were gated out, and MAIT cells were identified as CD3 + CD4-Vα7.2 + CD161 + T cells. The remaining non-MAIT T cell fraction was used to identify classical CD8 T cells (see Fig. 1A for gating strategy). Absolute MAIT, CD8 and CD4 T cell counts were calculated based on absolute lymphocyte counts at inclusion and their respective percentages of live CD3 + lymphocytes determined by flow cytometry.

### Statistical analyses

Statistical analyses were performed using Tibco Statistica (Version 13), Prism (Version 6) and R software. For comparison of medians Mann–Whitney U Test was used. Multi-variable logistic regression analyses were performed with hospitalizations (any vs none) as dependent variable and MAIT cell-related parameters as independent variables, correcting for known determinants of hospitalizations, i.e. FEV_1_ (% predicted) and GOLD 2017 group (A–D), by adding those two as additional independent variables into the models. In order to assess a potential confounding effect of use of inhaled corticosteroids (ICS), ICS use (any vs none) was added as additional independent variable into the multi-variable logistic regression models. Given the limited number of subjects, no additional independent variables were included in the multi-variable logistic regression analyses. For correlation analyses the Spearman test was used. All tests were two-sided and p < 0.05 was considered as significant.

## Results

**Fig. 2:**
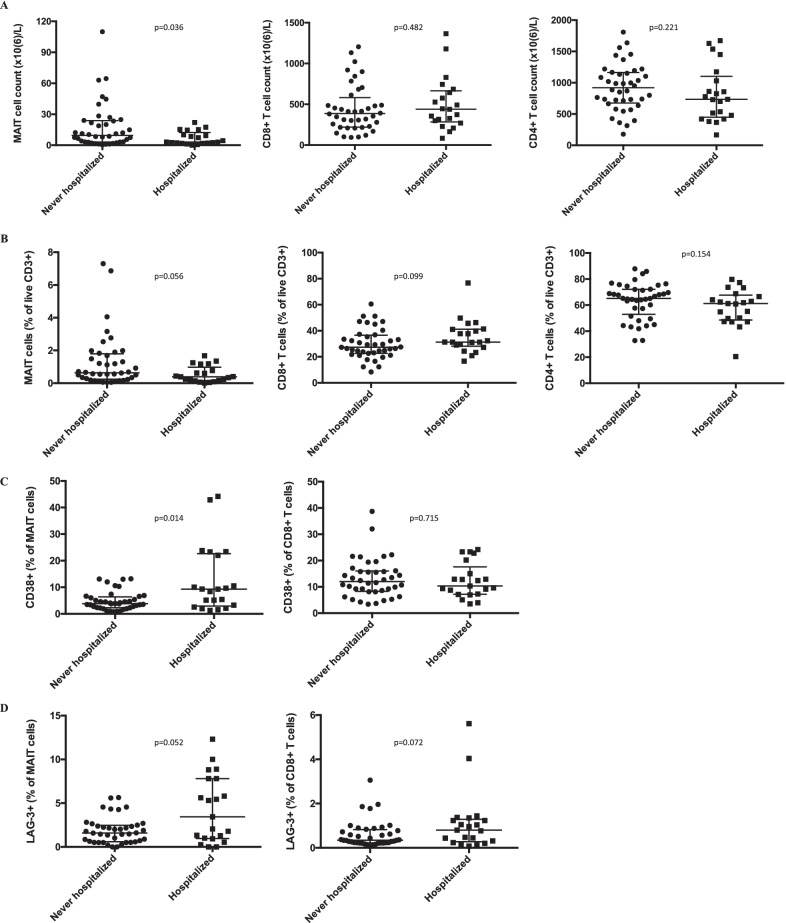
MAIT cell count and expression of the activation marker CD38 are associated with the risk of hospitalization. MAIT cell count, CD8 T cell count, CD4 T cell count (**A**), MAIT cell percentage, CD8 T cell percentage, CD4 T cell percentage (**B**), CD38 expression on MAIT cells, CD38 expression on CD8 T cells (**C**), LAG-3 expression on MAIT cells, and LAG-3 expression on CD8 T cells (**D**) in COPD subjects who required hospitalization during the 3-year follow-up (n = 21), compared to subjects who were not hospitalized (n = 40). Mann–Whitney U Test in all. Bars represent median with interquartile range

All patients were recruited, and blood was sampled, when in stable disease phase. The patients were followed for 3 years to record all-cause hospitalizations. We first compared patients who required hospitalization at one or more occasions during the 3-year follow-up (n = 21, referred to as “hospitalized” patients) with patients who never got hospitalized during this time period (n = 40, referred to as “never hospitalized” patients). Interestingly, hospitalized patients had lower MAIT cell count at study inclusion, i.e. in a stable disease phase, compared to never hospitalized subjects (p = 0.036, Fig. 2A). In contrast, hospitalized and never hospitalized patients did not differ in CD8 or CD4 T cell counts. There was also a trend towards lower MAIT cell percentage in hospitalized patients compared with never hospitalized subjects (Fig. 2B). Next, we investigated whether MAIT cells in hospitalized patients were phenotypically different from those in the never hospitalized group. MAIT cells in hospitalized subjects showed a more activated phenotype with higher CD38 expression, and there was a trend towards higher lymphocyte activation gene 3 (LAG-3) expression (Fig. 2C and D). Expression of CCR5, programmed cell death protein 1 (PD-1), T cell immunoreceptor with Ig and ITIM domains (TIGIT), T cell immunoglobulin mucin 3 (TIM-3), CD8, CD56, HLA-DR, Ki-67, granulysin, promyelocytic leukemia zinc finger protein (PLZF), T-box transcription factor TBX21 (T-bet) and the transcription factor T cell factor 1 (TCF-1) by MAIT cells or by conventional CD8 T cells did not differ between hospitalized and never hospitalized patients (Fig. 1B, and data not shown).

We next sought to understand whether the state of the circulating MAIT cell compartment may add predictive value beyond the patients’ FEV_1_ and GOLD 2017 group. We performed multi-variable logistic regression analysis with hospitalizations (any vs none) as dependent variable, and FEV_1_ (% predicted), GOLD 2017 group (A–D) and quantity or activation of MAIT cells or conventional T cells as independent variables. The MAIT- and T-cell-related variables were entered into the model each separately. Interestingly, MAIT cell count, CD38 expression on MAIT cells, and LAG-3 expression on both MAIT and CD8 T cells were all independently associated with the risk of hospitalization (Table 2).

**Table 2:** Results of multi-variable logistic regression analyses with hospitalizations as dependent variable

Independent variable	Regression coefficient	Lower CL 95%	Upper CL 95%	p-value
MAIT cells (% of live CD3 +)	− 0.958	− 1.990	0.074	0.069
CD8 T cells (% of live CD3 +)	0.042	− 0.008	0.092	0.097
CD4 T cells (% of live CD3 +)	− 0.035	− 0.079	0.008	0.113
MAIT cell count (× 10^6^/L)	− 0.074	− 0.148	0.000	0.049
CD8 T cell count (× 10^6^/L)	0.001	− 0.001	0.003	0.562
CD4 T cell count (× 10^6^/L)	− 0.001	− 0.003	0.000	0.114
CD38 + (% of MAIT cells)	0.212	0.068	0.356	0.004
CD38 + (% of CD8 T cells)	− 0.008	− 0.092	0.075	0.844
LAG-3 + (% of MAIT cells)	0.639	0.255	1.022	0.001
LAG-3 + (% of CD8 T cells)	1.013	0.155	1.871	0.021

Multi-variable logistic regression analysis with hospitalizations (any vs none) as dependent variable, and FEV_1_ (% predicted), GOLD 2017 group (A–D) and quantity or activation of MAIT cells or classical T cells as independent variables. The MAIT- and T-cell related variables were entered into the model each separately. N = 61 in all

*CL* confidence level, *FEV*_*1*_ forced expiratory volume in 1 s, *GOLD* Global Initiative for Chronic Obstructive Lung Disease, *LAG-3* lymphocyte-activation gene 3, *MAIT* mucosal-associated invariant T

**Fig. 3:**
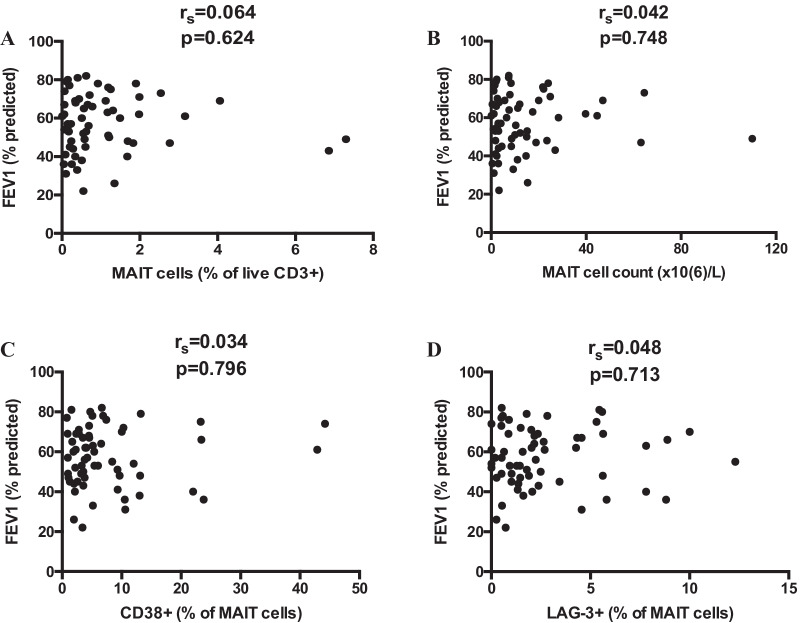
MAIT cell level and phenotype do not correlate with lung function. Relationship between MAIT cell percentage among the live CD3 + population (**A**), MAIT cell count (**B**), CD38 expression on MAIT cells (**C**), LAG-3 expression on MAIT cells (**D**), and forced expiratory volume in 1 s (FEV1) in the studied COPD cohort. Spearman correlation analysis. N = 61 in all. r_s_: correlation coefficient; p: p-value

Circulating MAIT cell levels were previously reported to be significantly lower in patients with moderate to severe COPD, compared with patients with mild COPD [30]. In contrast, in our COPD cohort the FEV_1_ measure did not correlate with MAIT cell count, MAIT cell frequency, or CD38 or LAG-3 expression on MAIT cells (Fig. 3). This was in line with the result of the multi-variable analyses, indicating that MAIT cells do not simply mirror the severity of airflow obstruction. Instead, MAIT cells may reflect a FEV_1_-independent immunological dimension of COPD, independently associated with the risk of all-cause hospitalization.

Hinks et al. previously observed that COPD patients treated with ICS had lower MAIT cell frequency in peripheral blood, compared with steroid-naive COPD patients [31]. We therefore investigated whether ICS use could influence the associations between MAIT cells and hospitalizations seen in our COPD cohort, and added ICS use (any vs none) as an additional independent variable in the multi-variable logistic regression analysis models. Hospitalizations were more likely in patients with higher CD38 and LAG-3 expression on MAIT cells independent of treatment with ICS (Table 3).

**Table 3:** Multi-variable logistic regression models with hospitalizations as dependent variable, evaluating effect of ICS (N = 61 in all)

Independent variables	Level of effect	Regression coefficient	Lower CL 95%	Upper CL 95%	p-value
Model 1					
Intercept	n.a	4.988	2.176	7.800	0.001
Baseline FEV_1_ (% predicted)	n.a	− 0.007	− 0.053	0.040	0.775
MAIT cell count (× 10^6^/L)	n.a	− 0.061	− 0.135	0.013	0.108
GOLD 2017 group	1 (group A)	− 5.442	− 6.539	− 4.346	< 0.001
GOLD 2017 group	2 (group B)	− 5.081	− 6.097	− 4.064	< 0.001
GOLD 2017 group	3 (group C)	13.412	n.a	n.a	n.a
ICS use (yes 1/no 0)	n.a	− 0.649	− 1.411	0.113	0.095
Model 2					
Intercept	n.a	3.562	0.400	6.724	0.027
Baseline FEV_1_ (% predicted)	n.a	− 0.016	− 0.065	0.033	0.526
CD38 + (% of MAIT cells)	n.a	0.221	0.076	0.367	0.003
GOLD 2017 group	1 (group A)	− 5.530	− 6.644	− 4.417	< 0.001
GOLD 2017 group	2 (group B)	− 5.957	− 7.052	− 4.862	< 0.001
GOLD 2017 group	3 (group C)	14.571	n.a	n.a	n.a
ICS use (yes 1/no 0)	n.a	− 0.815	− 1.737	0.108	0.084
Model 3					
Intercept	n.a	1.427	− 2.983	5.838	0.526
Baseline FEV_1_ (% predicted)	n.a	0.009	− 0.053	0.070	0.782
LAG-3 + (% of MAIT cells)	n.a	0.889	0.321	1.457	0.002
GOLD 2017 group	1 (group A)	− 7.986	− 10.033	− 5.938	< 0.001
GOLD 2017 group	2 (group B)	− 5.387	− 6.641	− 4.113	< 0.001
GOLD 2017 group	3 (group C)	16.024	n.a	n.a	n.a
ICS use (yes 1/no 0)	n.a	− 1.537	− 2.756	− 0.317	0.014

*CL* confidence level, *FEV*_*1*_ forced expiratory volume in 1 s, *GOLD* Global Initiative for Chronic Obstructive Lung Disease, *ICS* inhaled corticosteroids, *LAG-3* lymphocyte-activation gene 3, *MAIT* mucosal-associated invariant T, *n.a.* not applicable

## Discussion

In the COPD context it is interesting to note that MAIT cells have a broad effector profile, strong tissue homing ability, and are highly abundant in the liver and lung [7]. They contribute to mucosal homeostasis and protection against bacterial pulmonary infections [12–14]. On the other hand, exaggerated MAIT cell activation and recruitment to the airways might be involved in the immunopathogenesis of severe coronavirus disease 2019 [20, 21]. MAIT cells can also display profibrogenic activity [32], and a potentially pathogenic role of MAIT cells in inflammatory diseases has been reported [33, 34]. CD38 is a marker of T cell activation [35]. CD38 expression by sinonasal MAIT cells correlates with disease severity in patients with eosinophilic rhinosinusitis [36]. Peripheral blood MAIT cells in patients with chronic hepatitis B express higher levels of CD38 [37]. LAG-3 is a co-inhibitory receptor that is up-regulated on activated T cells [38]. This receptor was proposed to be a part of the exhausted and anergic signature of MAIT cells exposed to superantigens [39]. In our COPD cohort, MAIT cell count in peripheral blood, and expression of CD38 and LAG-3 on MAIT cells were associated with the risk of all-cause hospitalization. We speculate that COPD lung disease progression might be associated with enhanced MAIT cell activation, and recruitment of MAIT cells to the COPD airways. This process might possibly be driven by the accentuated chronic inflammation that characterizes COPD, and/or by repeated acute exacerbations and other acute clinical conditions that are associated with enhanced inflammation in COPD subjects. Whether the exhausted state of MAIT cells contributes to increased infection susceptibility and higher risk of acute exacerbations and therewith represents one of the underlying drivers for hospitalizations in COPD needs to be further investigated. Collectively, even though this is essentially a cross-sectional study with longitudinal follow-up, our findings suggest that MAIT cells may play a role in the pathophysiology of the chronic inflammatory response and tissue remodeling in the COPD lung.

A potential limitation of our study is the focus on all-cause hospitalizations instead of hospitalizations due to COPD exacerbation. However, in this data set majority of the hospitalizations were COPD related, so we used the broader definition to avoid classification errors. Another potential limitation is that we only recorded MAIT cell data at a single time-point at baseline when the patients were in a stable phase. It is possible that the cross-sectional nature of the study thus prevented us from detecting interesting changes over time. Finally, this type of study design can suffer from the potential issue of selection bias. Future studies examining MAIT cells during the time of exacerbations may provide additional insights into the role of these cells in COPD.

## Conclusions

In summary, this study indicates that MAIT cell count and expression of the activation markers CD38 and LAG-3 on MAIT cells in peripheral blood of COPD patients are associated with the risk of hospitalization during a 3-year follow-up independently of FEV_1_ and GOLD 2017 group. Even though this is an exploratory study, and the results need to be confirmed in an independent validation cohort, our findings support the hypothesis that MAIT cells might reflect a novel FEV_1_-independent immunopathological dimension in the complexity of COPD. Moreover, this study defines MAIT cells as a component of cellular immunity with prognostic potential in COPD, superior to that of conventional T cells. The potential implication of MAIT cells in COPD deserves further investigation to assess whether MAIT cells act here solely as a surrogate biomarker, or if MAIT cells represent an important causal player in the COPD pathogenesis.

## Supplementary Information

**Additional file 1.** Staining panels.

## Data Availability

The datasets used and/or analyzed during the current study are available from the corresponding author on reasonable request.

## Abbreviations

CAT: COPD Assessment Test
CCR5: C–C chemokine receptor type 5
COPD: Chronic obstructive pulmonary disease
FEV_1_: Forced expiratory volume in 1 s
FVC: Forced vital capacity
GOLD: Global Initiative for Chronic Obstructive Lung Disease
ICS: Inhaled corticosteroid
LAG-3: Lymphocyte-activation gene 3
MAIT: Mucosa-associated invariant T
MLogR: Multi-variable logistic regression
MR1: Major histocompatibility complex class I-related gene protein
PD-1: Programmed cell death protein 1
PLZF: Promyelocytic leukemia zinc finger
SVC: Slow vital capacity
T-bet: T-box transcription factor TBX21
TCF-1: T cell factor 1
TIGIT: T-cell immunoreceptor with Ig and ITIM domains
TIM-3: T-cell immunoglobulin mucin-3
